# An empirical bioethical examination of Norwegian and British doctors' views of responsibility and (de)prioritization in healthcare

**DOI:** 10.1111/bioe.12925

**Published:** 2021-08-31

**Authors:** Jim A. C. Everett, Hannah Maslen, Anne‐Marie Nussberger, Berit Bringedal, Dominic Wilkinson, Julian Savulescu

**Affiliations:** ^1^ School of Psychology University of Kent Canterbury UK; ^2^ Oxford Uehiro Centre for Practical Ethics, Faculty of Philosophy University of Oxford Oxford UK; ^3^ Department of Experimental Psychology University of Oxford Oxford UK; ^4^ LEFO, Institute for Studies of the Medical Profession Oslo Norway; ^5^ John Radcliffe Hospital Oxford UK; ^6^ Murdoch Children's Research Institute Melbourne University Melbourne Victoria Australia; ^7^ Melbourne Law School Melbourne Victoria Australia

**Keywords:** attitude of health personnel, decision making, health priorities/ethics, healthcare rationing/ethics, Norway, UK

## Abstract

In a world with limited resources, allocation of resources to certain individuals and conditions inevitably means fewer resources allocated to other individuals and conditions. Should a patient's personal responsibility be relevant to decisions regarding allocation? In this project we combine the normative and the descriptive, conducting an empirical bioethical examination of how both Norwegian and British doctors think about principles of responsibility in allocating scarce healthcare resources. A large proportion of doctors in both countries supported including responsibility for illness in prioritization decisions. This finding was more prominent in zero‐sum scenarios where allocation to one patient means that another patient is denied treatment. There was most support for incorporating prospective responsibility (through patient contracts), and low support for integrating responsibility into co‐payments (i.e. through requiring responsible patients to pay part of the costs of treatment). Finally, some behaviours were considered more appropriate grounds for deprioritization (smoking, alcohol, drug use)—potentially because of the certainty of impact and direct link to ill health. In zero‐sum situations, prognosis also influenced prioritization (but did not outweigh responsibility). Ethical implications are discussed. We argue that the role that responsibility constructs appear to play in doctors' decisions indicates a needs for more nuanced—and clear—policy. Such policy should account for the distinctions we draw between responsibility‐sensitive and prognostic justifications for deprioritization.

## 1 INTRODUCTION

In a context of limited resources, public health authorities and medical health practitioners often have to make difficult decisions about how to allocate funds, treatments, and very scarce resources, such as organs. The current COVID‐19 pandemic has brought this into sharp focus with decisions having to be made about how to allocate ventilators to patients in respiratory failure.^1^ In an ideal world with unlimited resources, everybody would have immediate access to the best treatment available. We do not, however, live in such a world. Allocation of resources to certain individuals and conditions inevitably means fewer resources allocated to other individuals and conditions. Correspondingly, a central task in bioethics has been to systematically examine the kinds of principles that could be ethically justifiable in choosing how to allocate such scarce resources.^2^

There is a vast amount of normative work in philosophy and bioethics exploring the concept of responsibility^3^ and the kinds of decisions and policies that could be ethically justifiable in allocating scarce resources.^4^ A variety of different proposals have been discussed, including polices that are to some extent sensitive to patients' moral responsibility for their ill heath, others that discriminate on the basis of the behaviours patients have engaged in, or on the basis of differential prognosis given lifestyle factors.^5^

Such proposals indeed rest on subtly different justifications, some of which invoke moral responsibility and some of which are adjacent but distinct. We distinguish between three responsibility‐sensitive and one prognostic allocation policy:

1. Policies that explicitly deprioritized patients who are (retrospectively) morally responsible for their role in causing their ill health would effectively penalize those patients in response to a perceived lesser claim on resources.
2. A ‘solidarity’ policy, according to which healthcare resources are a public good, could advocate ‘charging’ people to engage in unhealthy behaviour by asking them to share healthcare costs through co‐payment when such behaviour is statistically linked to the state of ill health requiring treatment. Such a policy may be neutral on individual patients' moral responsibility for those states of ill health in particular cases.
3. Policies that introduced lifestyle contracts, according to which patients sign up to forgo unhealthy behaviours could justify deprioritization in cases of breach of such contracts. In such cases, a (prospective) responsibility to forgo the unhealthy behaviours is not met.
*The above three policy types are ‘responsibility‐sensitive’ in some way*.
4. Finally, policies that deprioritized patients on the basis of worse prognosis might *appear* to be holding those patients responsible, but no such claim need in fact be made in order to argue that resources should be distributed to maximize health outcomes across the population.

*This final policy type is not responsibility‐sensitive, although it may appear so in cases where the prognosis is poor as a result of health‐affecting behaviour (such as smoking or over‐eating)*.

For all this normative work, however, there is comparatively little systematic investigation of how frontline healthcare professionals *do* descriptively think about responsibility in resource allocation cases.^6^

In this work we fill this gap, combining the normative and the descriptive by conducting an empirical bioethical examination of how both Norwegian and British doctors think about principles of responsibility in allocating scarce healthcare resources. First, we sought to shed light on how medical professionals think about personal responsibility and (de)prioritization in healthcare, both in terms of more abstract principles and judgements in specific, detailed medical dilemmas. Second, we sought to explore the degree to which such judgements might be sensitive to the national context in which the medical professionals work.

In achieving these aims, we take care not to presuppose a particular account of responsibility. We assume only that the concept of (moral) responsibility for an outcome (i.e. backwards looking responsibility) is a function of knowledge and control.^7^ While, therefore, other scholars have made philosophical distinctions between different species of moral responsibility (e.g. attributability, accountability, answerability^8^), we take a broad view that can include the various types of backwards responsibility as well as forward‐looking responsibility.^9^ In this way we take our lead from work in experimental philosophy and experimental bioethics,^10^ adopting a more bottom‐up approach to explore how healthcare professionals appear to *actually* think about responsibility and how they incorporate notions of personal responsibility in their beliefs about deprioritization.

In the same way, we do not take a position on whether people *should* be held responsible for certain acts when subject to potentially compulsive desires. The debate about whether people with addiction^11^ or mental illness more generally^12^ are fully responsible for engaging in some harmful acts, and whether this should be factored into (de)prioritization, remains a live ethical issue subject to much debate. Given this, we do not take a position in this paper. Rather, we descriptively explore what allocation decisions doctors actually do make in these cases—for example do they endorse diverting resources away from someone with addiction. We do not seek to address whether they are normatively justified in making these decisions. Healthcare professionals are unlikely to be as intimately familiar with philosophical distinctions between types of responsibility as philosophers are, and yet it is critical to understand how these people that are on the front line think about these issues. Further, our task here is to demonstrate that policymakers need to reach a position on the defensibility of responsibility‐sensitive policies, especially if it is the case that responsibility (or responsibility adjacent) factors are playing a role in the decisions made by healthcare professionals.

### 1.1 How do medical professionals think about personal responsibility and (de)prioritization?

With an increasing focus on personal responsibility in health emerging in public discourse, a small but growing body of work over the last decade has considered how laypeople think about responsibility and allocation of healthcare resources. In the Netherlands, for example, people tend to disagree with the idea that those with unhealthy lifestyles should benefit from the healthcare system without a financial arrangement,^13^ and in Australia people give a low priority in allocating healthcare resources to people perceived as ‘self‐harmers’ because of smoking, or heavy drinking.^14^ In the United States, one survey showed that the majority of participants believed that there should be higher health insurance premiums for smokers.^15^ On the other hand, surveys of a general British population have showed almost an equal split between those who agreed and those who disagreed with idea that the NHS should pay the healthcare costs related to risky behaviours, with wider consensus among respondents in relation to smoking, heavy drinking, and sedentary lives, than to overeating, or poor diets.^16^ Such studies have begun to provide valuable insight into how ordinary people think about personal responsibility and (de)prioritization, but there is much we still need to know.

Most importantly, we need to know how *practicing healthcare professionals* think about these issues. Healthcare professionals—and especially doctors—are on the front line of diagnosis and treatment, and therefore have significant impact on priorities in healthcare. Doctors routinely face decisions about referring patients for treatments, or are tasked with prioritizing attendance between multiple patients. Important insight into this has already been provided by a few studies. For example, as part of a broader survey on which areas of the healthcare system needed more funding, and how this should be financed, Werntoft and Edberg^17^ show that only a small minority of Swedish physicians (11%) believed that ‘If patients have caused their disease themselves they should pay for treatment’ (though a more recent focused survey of Swedish physicians found that 83.9% of doctors believed that a hip replacement surgery should be made conditional upon giving up smoking, suggesting that personal responsibility is being considered^18^). In previous work one of us (BB) showed that most Norwegian doctors disagree with the idea that healthcare priority should depend on the patient's responsibility for the disease,^19^ with recent studies replicating this among Portuguese physicians.^20^ Interestingly, in line with the findings of Miraldo et al.^21^ on laypeople, both Norwegian and Portuguese physicians were more likely to agree with deprioritization based on a patient's smoking or heavy drinking than they were based on a patient's diet or fitness.^22^

In this study we wanted to investigate how medical professionals think about personal responsibility and (de)prioritization in healthcare by supplementing previously‐developed questions^23^ with new questions that we designed to probe the phenomenon further. The existing studies have given insight into how doctors respond to general statements about responsibility (e.g. do they agree or disagree that ‘Healthcare priority should depend on the patient's personal responsibility for the disease’), and which factors doctors think should be taken into account for (de)prioritization (e.g. excessive alcohol consumption, obesity, smoking).

In our work we wanted to replicate this in a British sample while also including new measures that allowed us to gain a deeper understanding. For example, it remains unclear based on the existing work how much doctors' responses are based on perceptions of personal responsibility rather than prognosis. Bioethical debate has highlighted tension between personal responsibility and prognosis as justifications for treatment allocation,^24^ and some of the measures were designed to help clarify this. We examined doctors' responses to detailed vignettes describing patients varying in both personal responsibility and prognosis, and asking which patient should be treated.

### 1.2 Are there differences between countries with different regulations in how medical professionals think about personal responsibility and (de)prioritization?

Different distributive principles and frameworks are employed in different systems. In the United Kingdom, two overarching principles are employed: first, ‘equal opportunity of treatment for those in equal need’, and, second, a (broadly) utilitarian principle that directs spending and allocation to maximize the health benefits accrued across the population from that spending.^25^ These principles might be thought to be somewhat in tension,^26^ but together potentially preclude differential treatment of patients on any grounds other than therapeutic effectiveness.

As a solidarity‐oriented welfare state, the situation in Norway is not dissimilar to that of the United Kingdom. Within specialized healthcare, priorities in Norway are legally required to be based on the following three criteria: (a) severity of the disease, (b) benefit of treatment, and (c) cost‐effectiveness of the intervention.^27^ However, there are a couple of differences between the operation of the Norwegian system compared to the United Kingdom. For example, cost‐sharing, or ‘co‐payment’ is to some extent more prominent in Norway than the United Kingdom: In Norway, patients pay between €15 and 35 per GP consultation and out‐patient visit, up to a ceiling of €240 per year. Through these charges, the Norwegian Ministry of Health aimed to curb demand from people with minor healthcare problems, reduce the growth in public spending and free up resources for high‐priority areas.^28^ However, to date, such cost‐sharing in Norway has not explicitly differentiated between patients on the basis of factors such as responsibility. No such charges exist for these particular NHS services in the United Kingdom (but in both the United Kingdom and Norway patients co‐pay for prescription medication and dental care).

The respective official positions on the (ir)relevance of a patient's personal responsibility to their claim to resources differ to some extent between the United Kingdom and Norway. In Norway, whether responsibility should be taken into account in prioritization has been explicitly considered—and rejected—by two governmental committees (in 1997 and 2014). Indeed, while Norwegian legislation explicitly defines the criteria upon which priorities should be made, patient responsibility is not among them. In the United Kingdom, although no official statement has been made to explicitly *permit* deprioritization on the basis of personal responsibility, one's personal responsibility for one's health has been highlighted in official statements. For example:

The NHS belongs to all of us. There are things that we can all do for ourselves and for one another to help it work effectively, and to ensure resources are used responsibly… [including recognising] that you can make a significant contribution to your own, and your family's, good health and well‐being, and take personal responsibility for it’ (The NHS Constitution, 2015^29^).

Further, there are practices and policies in the United Kingdom that might appear to give at least some weight to personal responsibility. For example, in particular contexts, patients are asked to sign a contract agreeing to make changes to their lifestyle—for example to practice healthier nutrition or stop smoking.^30^ Indeed, a 2016 report from the UK‐based Royal College of Surgeons found that over one in three Clinical Commissioning Groups (CCGs) in England were denying or delaying routine surgery to patients—such as hip and knee replacements—until they stop smoking or lose weight, in contravention of national clinical guidance.^31^ Such policies have rapidly made their way into public consciousness, sparking intense public debate about the fairness of such policies, and whether they are a necessary and justifiable policy or instead just a punitive measure to punish individuals for making ‘bad’ lifestyle choices.^32^

Importantly, however, official guidance from the NHS Commissioning Board^33^—like in Norway—explicitly forbids prioritizing based on personal responsibility, stating that ‘Individual patients or groups should not be unjustifiably advantaged or disadvantaged on the basis of [many things, including] lifestyle’.

In summary, we therefore note the following differences and similarities:

1. Both systems adopt a primary guiding principle of ensuring sufficient cost‐effectiveness in healthcare.
2. The relevance of personal responsibility for healthcare resource allocation has been explicitly considered and rejected in Norway but not in the United Kingdom.
3. There are more instances of (responsibility‐insensitive) cost‐sharing in Norway than in the United Kingdom.
4. Patients in the United Kingdom but not in Norway are sometimes, but not always, declined surgery unless they stop smoking or lose weight.

### 1.3 Aims of this paper

We sought to understand how medical professionals in Norway and the United Kingdom think about personal responsibility and (de)prioritization in healthcare. Given the national healthcare systems' similarities and differences, we aimed to compare the views of doctors in Norway and the United Kingdom regarding various ways in which personal responsibility could affect prioritization.

We present and discuss results from surveys of practitioners in both countries to assess differences of opinions, and whether these are in or out of line with official directives and practices. Our survey and discussion also enable us to tease apart different ways in which responsibility might be taken into account, and which of these, if any, are favoured by practitioners.

We hypothesized that practitioners in Norway would be less in favour of responsibility‐sensitive allocation in general.

## 2 METHOD

### 2.1 Participants

For the British data, participants were recruited via Wilmington Healthcare in December 2018. Qualified medical professionals in the United Kingdom (England, Scotland, Wales, and Northern Ireland) who were part of a large survey mailing list (*n* = 8,500) were invited to participate, and participants completed the survey online. Four hundred and ninety‐nine participants (response rate: 6%) consented to take part, and for the purposes of analyses we included only participants who answered at least 50% of the questions. After excluding 71 participants, we were left with a final sample of 428 British medical professionals, including both general practitioners (27% of the overall sample) and hospital specialists (73%). The majority of our sample were female (61.2%) and aged between 45 and 64 (81%).

For the Norwegian data, participants belong to a representative panel of 1,500–2,000 doctors, established in 1994 and surveyed biannually. In 2014–2015 the panel consisted of 1,545 doctors, working in all health services. The participants completed the survey as a postal questionnaire. One thousand one hundred and fifty‐eight of the 1,545 invited responded, leaving a response rate of 75%. As for the UK data, for the purposes of analyses we used a cut‐off criterion of requiring participants to answer at least 50% of the questions to be included in the analysis. This left a final sample of 1,141 participants, with the majority being male (57.6%) and between 45 and 64 (50.2%). Further details on the collection of the Norwegian data can be seen in Bringedal et al.^34^

### 2.2 Measures

#### 2.2.1 General responsibility statements

The first set of questions were five general statements concerning the extent to which participants agreed that personal responsibility should be taken into account for healthcare. Participants indicated their response with five options: *1 = disagree completely, 2 = disagree, 3 = neutral, 4 = agree*, and *5* = *agree strongly*.

**Table 1:** Vignette text

Vignette 1	‘You are the treating doctor of two patients. Patient A is long standing alcoholic who has given up drinking and has been sober for the last year. He has end stage alcoholic cirrhosis. Patient B has primary biliary cirrhosis with end stage cirrhosis. Both have had life threatening events in the last months and both are in their forties with no children and are single. They have both been waiting for a transplant for an equal amount of time. A liver has become available that is an ABO match for either patient. Which patient should receive the transplant?’
Vignette 2	‘You are in a remote location called to two emergencies. You can only attend one immediately and 2 ambulances are on their way but will take 20 mins to arrive to the patients. Patient B was helping her husband saw wood and her hand was caught in the electric saw—her hand has been severed and she is bleeding heavily, possibly fatally. Patient A is a long‐standing patient in the clinic. She has a history of recurrent self‐harm. You receive a call from her husband. Patient A has slashed her wrists and is bleeding heavily, perhaps fatally. Which patient should you attend to?’
Vignette 3	‘Patient A is a life‐long smoker. He grew up on a farm and all his family smoked. He has end stage emphysema and requires a lung transplant to survive. He is currently smoking. He is 45 and in otherwise good health. Patient B is a non‐smoker, but has end stage emphysema from alpha 1 antitrypsin deficiency. He is 40 but in poor health and had coronary artery bypass grafts last year. You can refer only one patient for lung transplantation next week. Who do you refer?’

#### 2.2.2 Conditions for allocation

The second set of questions looked at which specific factors participants thought should imply de‐prioritization. Participants were given a list of nine risk‐factors: (a) overweight/obesity, (b) smoking, (c) excessive alcohol consumption, (d) drug abuse, (e) lack of physical exercise, (f) high risk sports leading to injury or disease, (g) poor quality nutrition, (h) combination of the factors, and (i) violation of contract of changed lifestyle. For each factor, participants indicated either ‘Yes’ the factor should result in de‐prioritization, ‘No’ it should not, or that they did not know.

#### 2.2.3 Vignettes

Third, we gave participants a set of three vignettes, taken from an existing Norwegian project used in previous research^35^ and translated into English. Each contained brief descriptions of a hypothetical clinical scenario in which there are limited resources and only one of two patients can be helped (see Table 1). These vignettes were chosen for two key reasons: first, they have already been used in Norwegian samples over the last decade; and second, by using three scenarios we could begin to tease apart the potential justifications, which might draw on either perceptions of responsibility or perceived prognosis (or both). For a longer discussion of the relevant ethical features, see Section 4.1.3 and Table 5.

## 3 EMPIRICAL ANALYSIS

To analyse the data, we conducted a series of regression models (linear regression for the questions scored on a scale; multinomial regression for the questions with categorical response options), entering in country as a predictor and controlling for participant age and gender.

### 3.1 Comparative results (United Kingdom and Norway)

#### 3.1.1 General responsibility statements

**Figure 1:**
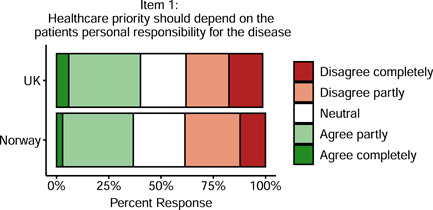
Support for taking into account patients’ personal responsibility into healthcare priority

Doctors in Norway and the United Kingdom had similar views on whether healthcare priority should depend on the patient's personal responsibility for the disease (see Table 2), with no significant difference in agreement between the two countries, *p* = .62. Forty per cent of British doctors and 37% of Norwegian doctors agreed that healthcare priority should depend on the patient's personal responsibility for the disease, while 37% of British and 38% of Norwegian doctors disagreed (see Figure 1).

**Table 2:** General responsibility questions

		Disagree (%)	Neutral (%)	Agree (%)
1. ‘Healthcare priority should depend on the patient's personal responsibility for the disease’	United Kingdom	36.68	21.73	40.19
	Norway	38.48	24.71	36.72
2. ‘Access to expensive treatment should depend on the patient's personal responsibility for the disease’	United Kingdom	39.49	20.33	39.25
	Norway	34.60	22.68	42.63
3. ‘Access to scarce organ transplants should depend on the patient's personal responsibility for the disease’	United Kingdom	33.64	21.50	44.39
	Norway	27.27	20.39	52.07
4. ‘Lower priority should be allotted to patients who violate a contract of changes in lifestyle’	United Kingdom	19.39	18.46	61.45
	Norway	24.54	28.68	46.51
5. ‘A patient who is responsible for the disease should pay additional co‐payments’	United Kingdom	57.94	21.73	20.09
	Norway	67.43	22.77	9.27

Agreement was also comparable across countries on whether access to expensive treatment should depend on the patient's personal responsibility for the disease, *p* = .066. Thirty‐nine per cent of British doctors and 43% of Norwegian doctors agreed, while 39% of British and 35% of Norwegian doctors disagreed.

**Figure 2:**
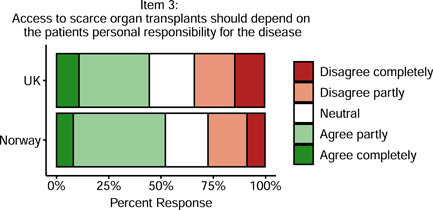
Support for taking into account patients' personal responsibility for their illness when deciding access to organ transplants

There was a non‐significant but trend‐level difference between the British and Norwegian doctors in their views on responsibility and organ transplantation, *p* = .05, with 52% of Norwegian doctors compared with 44% of British doctors agreeing that access to scarce organ transplants should depend on the patient's personal responsibility for the disease (see Figure 2).

British doctors were more likely than Norwegian doctors to agree that lower priority should be allotted to patients who violate a lifestyle change contract (61% vs. 47%), *p* < .0001, while doctors in Norway were more likely to be neutral (29%) than UK doctors (18%).

**Figure 3:**
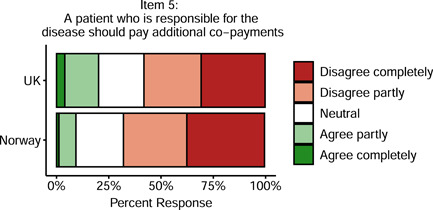
Support for having patients who are responsible for their disease pay additional co‐payments

British doctors were slightly more likely than Norwegian doctors to agree that patients who are responsible for their disease should pay additional co‐payments (20% vs. 9%), *p* < .0001. In both countries, however, the majority disagreed: 58% in the United Kingdom, and 67% in Norway (see Figure 3).

#### 3.1.2 Conditions for allocation

Next, we looked at which factors should lead to a lower priority for patients' treatment where present.

**Figure 4:**
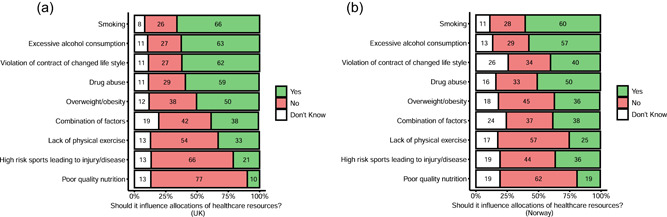
(a) Factors to be taken into account for allocation of healthcare (UK). (b) Factors to be taken into account for allocation of healthcare (Norway)

A majority of doctors in both the United Kingdom and Norway endorsed the idea that smoking, drug abuse, and excessive alcohol should be taken into account for deprioritization of resource allocation. In contrast, a majority of doctors in both the United Kingdom and Norway rejected taking into account lack of physical exercise, participation in high risk sports, and poor quality nutrition (Figure 4).

Next, we looked at whether there were differences in responses (yes vs. no vs. don't know) as a function of country, controlling for the influence of age and gender. We found significant differences between countries for weight, *χ*²(2) = 22.54, *p* < .001, smoking, *χ*²(2) = 7.11, *p* = .029, drug use, *χ*²(2) = 9.13, *p* = .01, lack of exercise, *χ*²(2) = 9.75, *p* = .007, violation of contract of changed lifestyle, *χ*²(2) = 75.90, *p* < 0.001, involvement of high risk sports, *χ*²(2) = 53.03, *p* < .001, and poor‐quality nutrition, *χ*²(2) = 29.13, *p* < .001. There was no difference in responses as a function of country for alcohol, *χ*²(2) = 3.99, *p* = .14, and a combination of factors, *χ*²(2) = 5.68, *p* = .059.

Looking at those factors for which there was a significant difference in overall responses by country, we focused in on the most important comparison: those doctors in each country who endorsed taking the factor into account compared to those who rejected taking it into account. We found that British doctors were more likely than Norwegian doctors to say that weight (*p* < .001), change of lifestyle contract (*p* < .001), and lack of exercise, (*p* = .012), *should* be taken into account than it should not.

In contrast, Norwegian doctors were more likely than British doctors to say that poor quality nutrition (*p* < .001), and high risk sports (*p* < .001), should be taken into account.

There was no difference between Norwegian and British doctors in how much they endorsed versus rejected taking into account smoking, with the overall difference being driven by Norwegian doctors being more likely to say they did not know (*p* = .03).

#### 3.1.3 Vignettes

Our first vignette asked participants about liver transplantation to a patient with primary biliary cirrhosis (‘Patient B’) or one with alcoholic liver disease (‘Patient A’: see Table 1 for full text, and Table 3 for responses). We found that participants were more likely to indicate that the patient with primary biliary cirrhosis—the relatively less personally responsible one—should receive the liver transplant. We found that Norwegian doctors were especially likely to choose the less personally responsible patient over the patient with alcoholic liver disease (50% vs. 1%), compared to British doctors (37% vs. 4%) (*p* < .001).

Our second vignette dealt with a case of emergency injury, and whether to attend to a patient involved in a chainsaw accident (‘Patient B’) or one who has self‐harmed (‘Patient A’). In both Norway (72%) and the United Kingdom (66%) the majority of participants indicated that they should attend to Patient B—the relatively less personally responsible patient, and there was no significant difference between the two countries in the proportion choosing Patient B over Patient A.

Our third vignette dealt with a case of emphysema, and whether to recommend for a lung transplantation a patient who was a life‐long smoker but in good health (‘Patient A’), or a patient who is a non‐smoker but in poor health (‘Patient B’). Overall, in both countries the majority of participants indicated that they should attend to Patient B—the relatively less personally responsible patient (though the difference was not as great as in the previous two vignettes).

### 3.2 UK‐specific results

#### 3.2.1 Locus of responsibility

The vast majority of our participants felt that participants had a responsibility to both themselves (90.89%) and their families (82.71%), and more than two‐thirds thought that people have a responsibility to society to look after their health (69.16%).

**Table 3:** Vignette responses

	Patient A		Patient B		Toss a coin		Do not know	
	Norway	United Kingdom	Norway	United Kingdom	Norway	United Kingdom	Norway	United Kingdom
Vignette 1 (%)	1.41	4.21	50.49	36.68	22.24	26.87	24.54	32.01
Vignette 2 (%)	1.59	2.80	71.67	66.12	8.38	13.79	18.36	17.06
Vignette 3(%)	19.68	25.23	47.40	54.44	6.18	4.67	26.21	15.42

#### 3.2.2 Support for elective procedures

After looking at descriptive statistics for all the items (see Table 4), we used paired‐sample *t*‐tests to look at differences between the key pairs of items. For both obese, *t*(416) = −17.28, *p* < .001, *d* = −0.083, and patients who smoke, *t*(421) = −17.76, *p* < .001, *d* = −0.084, doctors were significantly more likely to agree with *encouraging* patients to complete a period of changed lifestyle in which they stopped smoking/lost weight before they received elective procedures, than they were to agree that such elective procedures should simply be withheld until the patient has stopped smoking/lost weight. The same pattern was observed for obese women receiving IVF, *t*(416) = −15.91, *p* < .001, *d* = −0.067.

**Table 4:** Support for restricting elective procedures for patients who are obese or smoking

	Disagree (%)	Neutral (%)	Agree (%)
**Obesity**			
‘Elective surgical procedures should be withheld from obese patients until they have lost weight’	30.14	25.47	42.99
‘IVF should be withheld from obese patients until they have lost weight’	25.47	28.83	48.60
‘Obese patients should be encouraged to lose weight before they receive an elective procedure’	9.11	7.71	82.01
‘Obese patients should be encouraged to lose weight before they receive IVF’	8.64	9.58	81.31
**Smoking**			
‘Elective procedures should be withheld from patients who smoke until they quit smoking’	37.85	21.26	39.95
‘Patients who smoke should be encouraged to quit smoking before they receive an elective procedure’	12.15	8.41	78.27

There was a significant difference between obesity and smoking in the extent to which doctors endorsed both withholding treatment, *t*(418) = 3.76, *p* < .001, *d* = 0.14, and encouraging lifestyle changes, *t*(418) = 1.62, *p* < .001, *d* = 0.06, with doctors agreeing more for obese patients than they did patients who smoke.

### 3.3 Causes of unhealthy behaviour

Looking at participants' perceptions of the causes of unhealthy behaviour, our participants felt that nurture (i.e. environment and social learning: 40.16%) had the strongest influence on people's engagement in unhealthy behaviours, followed by free choice (i.e. personal decisions: 34.28%), with nature (i.e. biology and genetics: 26.58%) seen as the least impactful.

### 3.4 Allocation of scarce resources

In a dilemma about which child to recommend for an organ transplant—one child with Down syndrome, one without any underlying genetic condition‐, we found that most participants (73.13%) felt that whichever child was placed on the waiting list first should receive the heart, regardless of whether the child had Down syndrome or no underlying condition. A much smaller minority thought that the child with Down syndrome should have some, but a lower, chance of receiving the heart (16.12%), and very few participants felt that the child with Down syndrome should not receive the heart (6.07%).

### 3.5 Oxford Utilitarianism Scale

Finally, we looked at responses on the Oxford Utilitarianism Scale (OUS)^36^ and their relation to support for taking personal responsibility into account for prioritization. The OUS consists of two sub‐scales that dissociate individual differences in the ‘negative’ (permissive attitude toward instrumental harm) and ‘positive’ (impartial concern for the greater good, or impartial beneficence) dimensions of utilitarian thinking as manifested in the general population.

Looking first at the correlation of the OUS sub‐scales with the general responsibility statements, we found that endorsement of instrumental harm positively predicted taking personal responsibility into account across every item (all *p*s < .001). People who endorsed causing harm in order to bring about the greater good, that is, were more likely to agree that access to expensive treatment, and organ transplants should depend on the patient's personal responsibility, and that patients who are responsible for a disease should pay additional co‐payments. In contrast, impartial beneficence was only significantly correlated with one item, such that people who were more likely to endorse the impartial maximization of the greater good even at significant self‐sacrifice were more likely to think that patients who are responsible should pay additional co‐payments.

Next, we looked at the correlation of the OUS sub‐scales with the questions on elective procedures. Again, endorsement of instrumental harm positively predicted taking personal responsibility into account for elective procedures across every item (all *p*s < .05). Impartial beneficence was not significantly correlated with any item.

## 4 DISCUSSION

For the empirical component of this paper, we sought to understand how medical professionals in both Norway and the United Kingdom think about personal responsibility and (de)prioritization in healthcare. We compare the views of healthcare practitioners in Norway and the United Kingdom regarding various ways in which personal responsibility could affect prioritization.

Overall, in both countries, participants were fairly evenly split between agreeing and disagreeing that healthcare priority should depend on the patient's personal responsibility for the disease. In both countries, participants were more likely to agree that access to scarce organ transplants should depend on their personal responsibility for the disease, and in both countries, participants were more likely to disagree that a patient who is responsible for a disease should pay additional co‐payments. In both countries a majority of Norwegian and British doctors favoured deprioritization based on smoking, excessive alcohol and drug abuse, but a majority were opposed to using lack of physical exercise, high risk sports or poor nutrition. And in all three forced choice vignettes, for both countries, the largest proportion of doctors favoured giving treatment to a patient perceived not to be responsible for their illness. This was most prominent for a patient with injury due to self harm, with more than two‐thirds of respondents electing to prioritize a patient with accidental (rather than self‐inflicted) injury.

There were, however, some interesting differences. For example, Norwegian doctors were slightly more likely to agree that personal responsibility should influence organ allocation, while British doctors were more likely to support factoring responsibility into co‐payments. Similarly, a higher proportion of British doctors were in favour of making decisions sensitive to obesity or violation of a contract, while a higher proportion of Norwegian doctors supported deprioritization based on high risk sports or poor quality nutrition.

Our results are broadly consistent with previous work, both replicating and extending previous surveys looking at laypeople and physicians in different countries. Looking at the general responsibility statements, we found that 37% of British doctors and 38% of Norwegian doctors disagreed that ‘Healthcare priority should depend on the patient's personal responsibility for the disease’—a comparable proportion to the 34% found in Portuguese physicians^37^ when given the same question.

Both our United Kingdom and Norwegian sample were more likely to think that substance abuse and smoking should be taken into account in (de)prioritization than should be a lack of exercise and poor nutrition, mirroring previous findings in Norwegian physicians,^38^ Portuguese physicians,^39^ and both the British^40^ and Australian^41^ public. As for the responsibility statements, however, we found that Norwegian doctors appeared more willing to take personal responsibility into account in 2019 than they did in 2010: while 44% thought smoking should be taken into account in 2010, this increased to 60% in 2019; and while 38% thought alcohol abuse should be taken into account in 2010, this increased to 57% in 2019.

### 4.1 Ethical analysis

As noted in the Introduction, in this project we took an explicitly bottom‐up approach to understand how healthcare professionals think about responsibility and (de)prioritization in healthcare. We do not adopt a specific normative account, and there are number of potential justifications for responsibility‐sensitive allocation policies in healthcare. Our survey data captured doctors' attitudes regarding the relevance of patients' responsibility for resource allocation, but also allowed us to hypothesize and potentially distinguish between some of the justifications that could underlie their responses. It is highly unlikely that any of our respondents had such complex philosophical distinctions in mind, but we can draw some conclusions from our data that are relevant for ethical analysis.

Broadly, responsibility‐sensitive policies could be justified on four different grounds: *retrospective responsibility penalty, solidarity*/*cost‐internalization, breach of contract*, and *prognosis*.^42^ Before presenting a more focused ethical analysis from our data, we first briefly explain how each of these grounds might justify differential allocation of resources, before discussing the results of our survey in light of these.

*Retrospective responsibility penalty:* First, resource allocation might be thought to be justified on the grounds that the patient is morally responsible for their state of ill health, and therefore is less deserving of resources. The most robust version of this position would view patients as having failed in their moral responsibility to maintain their health, and therefore justify penalizing them both as a matter of desert and as a deterrent to others who might similarly fail in their moral responsibility to maintain their health (for discussion [and rejection] of this position^43^). This potential justification is retrospective in the sense that it holds patients responsible and penalizes them in response to their past behaviour. Such a justification would require that patients had sufficient control and knowledge to be morally responsible for their ill health. It could justify a wide range of policies that consistently prioritized patients who required treatment as a result of bad luck, regardless of their prognosis. Some have argued that this potential justification erroneously assumes that people have greater control over their behaviours than they actually do—such that in many instances it is not the case that patients are morally responsible for their ill health—and has been criticized for being overly moralizing even in cases where people do have sufficient control.^44^ In our survey, general statements one, two and three—‘Healthcare priority should depend on the patient's personal responsibility for the disease’, ‘Access to expensive treatment should depend on the patient's personal responsibility for the disease’, ‘Access to scarce organ transplants should depend on the patient's personal responsibility for the disease’—tap in to this sort of justification for (de)prioritization. Further, agreement with any of the statements regarding a locus of responsibility—whether to one's self, family, or society—implies perception of a moral duty to maintain good health. Attitudes towards whether failure in any of these perceived duties should bear on prioritization, however, can only be inferred from the general statements.

*Solidarity/cost‐internalization:* A second potential justification would hold that it is fair to require individuals to internalize the costs of their health‐affecting behaviour. Such a justification does not have to take a position on moral responsibility in individual cases, but instead posits that a solidarity‐based system, in which healthcare resources are a public good, is appropriately supported by ‘charging’ people to engage in unhealthy behaviour (such as through taxes on alcohol or cigarettes) or by asking them to share the costs of these behaviours when they lead to a claim on healthcare resources. Such a justification would still present some difficulty when it came to assessing who should share costs and who should not (i.e. difficulty establishing a causal connection between behaviour and illness), but would justify practices of cost‐sharing and co‐payment for certain behaviour‐disease combinations. This justification would not, for example, straightforwardly justify prioritization in the case of organ transplants, as it determines circumstances in which an individual has to contribute *extra* resources to the pool, rather than identifying any circumstances in which resources can be withheld. However, those who require organs as a likely result of engaging in unhealthy behaviour could be required to contribute financially, regardless of whether they are also deprioritized. General statement five—‘A patient who is responsible for the disease should pay additional co‐payments’—taps into this justification for differential practices.

*Breach of contract:* The third potential justification for responsibility‐sensitive allocation holds that, when an individual has committed to making feasible changes to their lifestyle to improve or maintain their health, they acquire a prospective responsibility to honour that commitment. A violation of a commitment or contract then justifies deprioritization regardless of relative prognosis compared to others who fulfil their lifestyle contract. Opportunities for asking patients to commit to lifestyle changes might arise, for example, where smoking or overeating is compromising their health. One of us (JS) has argued that there will be some ‘golden opportunities’ for patients to make changes in a cost‐ and risk‐free way, such as by switching from smoking tobacco to smoking electronic cigarettes.^45^ Prioritization on the basis of a violation of a lifestyle contract in these circumstances is therefore not subject to the same criticisms that apply to policies that hold patients retrospectively responsible, as at least the knowledge condition for responsibility will be more clearly fulfilled, and the patient has made a positive commitment in knowledge of the consequences of contractual violation. General statement four—‘Lower priority should be allotted to patients who violate a contract of changes in lifestyle’—taps into this sort of justification, as do the conditions for the allocation item ‘violation of contract to changed lifestyle’.

*Prognosis:* The final potential justification for differential allocation is not, in fact, sensitive to responsibility (strictly speaking,) but is nonetheless sensitive to patients' behaviour, insofar as it affects their prognosis. Allocation on the basis of prognosis essentially promotes efficient use of healthcare resources in a system. Treatments will be withheld, or patients deprioritized, in the event that their condition (and its expected trajectory, given the patient's overall health) is unlikely to improve sufficiently with treatment. Directing resources to the care that is expected to produce the most benefit (and away from care that is expected to produce no or little benefit) maximizes cost‐effectiveness. Allocation on the basis of prognosis could justify policies such as withholding some surgeries from smokers or obese individuals until or unless their behaviour changes to improve their prognosis, regardless of whether they sign a lifestyle contract. Items assessing support for elective surgery for smokers and obese patients tap into the view that some treatment should be withheld unless or until prognosis improves. It is important to note that deprioritization on the basis of prognosis overlaps with (i.e. would often lead to the same outcome as) deprioritization on the basis of a breach of lifestyle contract. Two patients could sign a contract that requires them to stop smoking. One patient may fulfil the contract, but have a very poor prognosis (perhaps due to many extra years of smoking), whilst the other violates the contract but has a much better prognosis. If fulfilment of lifestyle contract were the primary rationing criterion the patient who fulfilled the contract would be prioritized, if prognosis were the primary criterion it's possible that the patient who continues to smoke would be prioritized, due to much better health. Our vignettes were designed to vary responsibility and prognosis, to separate potential justifications.

#### 4.1.1 General responsibility statements

We asked Norwegian and UK doctors about the general relevance of responsibility to healthcare. The first three items (see Table 1), although not explicitly so described, tap into intuitions about holding people retrospectively responsible, in line with the first justification. They differ in the scarcity of the resource and therefore the extent to which they will involve zero‐sum treatment situations (in which one patient's treatment results in another's non‐treatment). Our data indicate that as the situation gets close to zero‐sum (from general healthcare, through expensive treatment, to scarce organs), doctors in both countries were more likely to say that treatment should depend on the patient's personal responsibility.

The fourth item captured doctors' views on deprioritization as a consequence of violating a lifestyle contract—a failure of prospective responsibility. Although doctors in Norway were less inclined than British doctors (perhaps as a consequence of UK doctors' greater familiarity with such contractual agreements), more doctors in both countries agreed that a patient's failure to fulfil a prospective responsibility should affect prioritization, than they did that healthcare priority should depend on the patient's (retrospective) personal responsibility for the disease. This aligns with claims one of us has defended regarding the greater permissibility of healthcare resource allocation that is sensitive to failures of prospective responsibility through violating lifestyle contracts, compared to policies that are sensitive to (apparent) retrospective responsibility for ill health.^46^

The fifth item captured doctors' views on patients paying additional co‐payments when they are responsible for their condition. More than half of the doctors in both countries were opposed to this, with Norwegian doctors even more likely to disagree than British doctors. Doctors may have been alert to the potential unfairness that could result from such a system: in such a system, those who are responsible but rich could access treatment more easily than those who are responsible but poor, unless there were means‐tested payment exemptions. Although also a topic of contention, cost‐internalization via high taxation on unhealthy behaviour (e.g. higher taxes on cigarettes, alcohol and sugary drinks) rather than treatment could be fairer.^47^ Proponents of such a view might argue that access to healthcare is more fundamentally important than access to unhealthy behaviours, and therefore that costs should attach to behaviour rather than treatments.

#### 4.1.2 Conditions for allocation

The second set of items asked doctors about their views regarding which behaviours or factors should ‘influence allocation priorities in healthcare, resulting in deprioritisation of patients' treatment where present’. Here, respondents were given more detail about the particular circumstances in which patients' responsibility—or at least their behaviour—might justify differential treatment. Responses to these items can instructively be compared to responses to the first general statement: in the present set of items, the precise basis on which a patient might be considered responsible is identified.

It is therefore striking that responses to some of these items demonstrated much greater agreement with deprioritization practices. In comparison, less than 40% agreed with the general statement on responsibility and prioritization.

Although there were some differences between the United Kingdom and Norway in the ranking of which factors garnered the most agreement, smoking, excessive alcohol consumption, and drug abuse all generated more than 50% agreement in both countries. There are a number of possible explanations for this finding. In order for an individual to be responsible for some outcome—in this case, ill health that requires treatment—the individual's acts (or failures to act) must have caused this outcome, and they must have had sufficient control over their acts and sufficient knowledge of the consequences. It may be that doctors perceive the causal link between ill health and each of smoking, drug use and excessive alcohol consumption, respectively, to be more certain (compared, e.g. to the causal link between ill health and lack of exercise). Further, doctors may perceive that facts about the detrimental effects of these behaviours are more widely and easily understood within the general population, and that it is easier for patients to abstain from these behaviours than it is to avoid poor quality nutrition, for example, which could be seen as closely connected to poverty.

However, on the basis of this data, we cannot rule out that, rather than having a justification on the basis of retrospective responsibility in mind, doctors were considering the likely prognosis of patients who engage in each behaviour. It could be that doctors believe that patients who smoke, drink or take drugs will benefit less from the relevant treatment than will individuals who do not exercise or have poor nutrition, for example. The former set of patients, but not the latter should, if efficiency is to be maximized, be deprioritized relative to patients who are expected to have better outcomes, if efficiency guides allocation.

Overall, it seems that when given more detail, doctors are more likely to support differential allocation, whether this is on the basis of retrospective responsibility or expected prognosis.

#### 4.1.3 Vignettes

In order to tease apart the potential justifications, which might draw on either responsibility or prognosis (or both), we presented three scenarios with different features, generating different combinations of responsibility and prognosis (see Table 5). We note that all these vignettes are zero‐sum scenarios. In line with the trends noted above, the zero‐sum nature and increased detail of the scenario seem to result in a perception of greater relevance of responsibility.

**Table 5:** Responsibility and prognosis across the vignettes

		Responsibility	Prognosis after treatment
**Vignette 1 (Cirrhosis)**	Patient A	High	Good (assuming sobriety)
	Patient B	Zero (no relevant behaviour; genetic condition)	Good
**Vignette 2 (Injury)**	Patient A	High	Poor (relapse likely)
	Patient B	Low (unlikely accident)	Good
**Vignette 3 (Emphysema)**	Patient A	High	Unclear (good health but still smokes)
	Patient B	Zero (no relevant behaviour; genetic condition)	Poor (in poor health)

In Vignette 1—Cirrhosis—Patient A could be perceived to be responsible and have a good prognosis after treatment (since ongoing sobriety is stipulated). Patient B is clearly not responsible, and potentially also has a good prognosis after treatment.

In Vignette 2—Injury—Patient A could be perceived to be responsible and have poor ultimate prognosis^48^ (it is stipulated that they have recurrent self‐harm). Patient B is not responsible (it was an unlikely accident) and potentially has a better prognosis than patient A.

In Vignette 3—Emphysema—Patient A could be perceived to be responsible. His prognosis to benefit from the transplant is uncertain since he still smokes. He is otherwise in good health. Patient B is not responsible, but could be expected to have poor prognosis (he is in poor health).

Overall the patient who could be perceived to be not responsible was preferred in every vignette. Since prognosis is potentially equivalent for both patients in Vignette 1, the preference for treating Patient B is likely to be a result of perceived responsibility only.

Doctors were most likely to prioritize the non‐responsible patient in the second vignette. This could represent the additive effect of perceived poorer prognosis. (However, we did not ask doctors for their reason for preferring Patient B. It is possible that clinicians had other factors in mind—for example, they may have felt that the patient who had amputated their hand needed more urgent attention. Alternatively, they may have had negative intuitions about the value of saving the life of a patient who recurrently self‐harms).

In the final vignette, prognosis potentially conflicted with responsibility, since Patient B potentially would have a worse outcome from the transplant. A larger proportion of respondents were inclined to prioritize Patient A in this vignette. However, Patient B was still preferred to Patient A, despite poorer prognosis.

These results show that as the items became more detailed regarding the patients' responsibility for their condition, doctors in both countries were more inclined to take responsibility into account. Indeed, in response to the vignettes, in circumstances of allocating indivisible goods (e.g. organs), the majority of doctors consistently allocated resources to the individual who was perceived to be less responsible for their need for resources. This was attenuated to some extent when prognosis was better for the patient who was responsible, but prognosis did not outweigh responsibility in any of the scenarios.

#### 4.1.4 Locus of responsibility

When individuals have a moral responsibility to do or refrain from doing something, this is almost always because their actions in this regard have implications for others. In the case of a responsibility to maintain one's health, we can ask which individual's, or which set of individuals', interests are affected by one's health‐affecting behaviour, and so to whom one ‘owes’ it to maintain one's health. Our results show that a clear majority of British doctors (90.89%) think that individuals have a responsibility—‘owe it’—to themselves to look after their health. It is a contentious view in ethics that there are moral duties that are purely self‐regarding. Rather than doctors agreeing that one has a self‐regarding moral duty, it might be that participants had something more akin to a ‘prudential responsibility’ in mind.^49^ Although we aren't *morally* required to look after our health for our own sake, we owe it to ourselves to do so in so far as it promotes our well‐being (and in so far as we care about our well‐being). However, even if there were to be a self‐regarding moral duty (and especially if it is non‐moral in nature), it would not be in keeping with a liberal state to ‘penalize’ individuals for failures in this regard.

A responsibility that is grounded in the duties we have to our families may, however, be straightforwardly moral in nature, especially where we have dependants (82.71% British doctors agreed that we owe it to our families to look after our health). When our lives are closely intertwined with others, our health can affect our relationships and our ability to fulfil our responsibilities to dependents. In so far as we should try to avoid impairing our ability to nurture our familial relationships and fulfil our familial obligations, we should attempt to avoid states of ill health that are deleterious. There will be disagreement about the strength and scope of such a duty, but even in this case, the plausibility of a state response via the mechanism of deprioritized healthcare seems an overreach into the private sphere.

Finally, 69.16% of British doctors agreed that individuals owe it to society to look after their health. This duty might be thought to be grounded in solidarity arrangements, seen in healthcare systems involving both rights and obligations.^50^ Since all are reliant on the limited system, avoidably depleting the system violates obligations of solidarity and therefore justifies penalty. The view that such a duty exists would therefore provide a justification for some instances of rationing. It would particularly support practices of cost‐sharing.

#### 4.1.5 OUS

British doctors who endorsed causing harm in order to bring about the greater good were more likely to agree that access to expensive treatment, and organ transplants should depend on the patient's personal responsibility, and that patients who are responsible for a disease should pay additional co‐payments. We interpret these results as indicating a preference for the expected deterrent effect that such policies would have, thus potentially increasing overall health and higher QALYs in the population, rather than any theoretical alignment of endorsement of instrumental harm with endorsement of a responsibility‐sensitive criterion of fairness/desert per se.

### 4.2 Limitations

There are, as with any empirical project, key limitations that must be considered. First, we had a low response rate from the British sample. While the survey was sent to 8,500 potential respondents in the United Kingdom, only 499 completed the survey, meaning it is difficult to generalize our conclusions to the population of British doctors as a whole. It is possible that those doctors who self‐selected to respond to the invitation to participate differ in some systematic way in their attitudes about responsibility from those who did not. That noted, we share this limitation with many previous studies, and the broadly similar pattern of results across the British and Norwegian (where response rate was very high) goes some way to alleviate this concern. Ultimately, we suggest that imperfect data are better than no data at all.

Second, our results are only able to show doctors' self‐reported attitudes, and we cannot speak to whether, and how much, doctors might explicitly or implicitly use personal responsibility when referring patients for treatment in their own practices. Further work should be done to examine the extent to which doctors act on their views, potentially in contravention of official policy.

Third, there is always the chance in comparative studies that translations can have subtly different meanings in different contexts. Our vignettes, in particular, were taken from an existing Norwegian project used in previous research^51^ and translated into English. It remains possible that our translation changed the meaning in the UK setting, and this could help explain some of the subtle country variations. That said, it is important to note that the English translations were vetted by one of the co‐authors (BB) who has used the Norwegian versions in previous work, and that none of the vignettes involved culturally‐specific conditions or backgrounds.

Finally, there is a need for further ethical analysis of potential policy implications if doctors' views were in fact reflected in policy—for example through the greater use of behavioural contracts in healthcare.

### 4.3 Conclusion

To our knowledge, this is the first international comparison of physician attitudes to prioritization and responsibility in healthcare. We found substantial overlap in the attitudes of British and Norwegian doctors, though also some differences. A large proportion of doctors in both countries supported including responsibility for illness in prioritization decisions. This finding was more prominent in zero‐sum scenarios, where allocation to one patient means that another patient is denied treatment. Doctors were also more inclined to agree with prioritization in more detailed and specific scenarios. There was most support for incorporating prospective responsibility (through patient contracts), and low support for integrating responsibility into co‐payments (i.e. through requiring responsible patients to pay part of the costs of treatment). Some behaviours were considered more appropriate grounds for deprioritization (smoking, alcohol, drug use)—potentially because of the certainty of impact and direct link to ill health. In zero‐sum situations, prognosis also influenced prioritization (but did not outweigh responsibility).

Our findings are important. In two solidarity‐based healthcare systems, doctors are of the view that responsibility should be taken into account, particularly for some behaviour‐illness combinations and where resources are particularly limited. Particularly in Norway, these views diverge from official policy and guidance regarding responsibility‐sensitive resource allocation. While healthcare professionals seem to take perceived responsibility and perceived prognosis into account, they do not seem to be informally adopting any specific account of responsibility or prognosis when making their decisions. This highlights the need for more clarity in official ethical guidance and policy. Whether or not doctors are justified in making responsibility‐sensitive allocation decisions, and what account of responsibility (if any) should be adopted is far more than a theoretical question: decisions in such cases have repercussions for people's lives. We have shown that responsibility‐like constructs are in play in some aspects of decision‐making in both the United Kingdom and Norway, despite differing (but somewhat vague) official positions. Policymakers must engage in ethical debate about how to more systematically structure, limit or encourage the decisional role that such factors are playing.

## CONFLICT OF INTEREST

The authors declare no conflict of interest.

## Footnotes

1 Savulescu, J., Vergano, M., Craxì, L., & Wilkinson, D. (2020). An ethical algorithm for rationing life sustaining treatment during the COVID‐19 pandemic. *British Journal of Anaesthesia, 125*(3), 253–258.

2 For example, see: ibid; Savulescu, J. (2018). Golden opportunity, reasonable risk and personal responsibility for health. *Journal of Medical Ethics, 44*(1), 59–61.

3 For example, see: Feinberg, J. (1970). *Doing and deserving: Essays in the theory of responsibility*. Princeton University Press; Fischer, J. M., & Ravizza, M. (1998). *Responsibility and control: A theory of moral responsibility*. Cambridge University Press; Smart, J. J. C. (1961). Free will, praise and blame. *Mind, 70*, 291–306; Strawson, P. (1962). Freedom and resentment. *Proceedings of the British Academy, 48*, 1–25; Watson, G. (1982). *Free will*. Oxford University Press; Levy, N. (2011). *Hard luck: How luck undermines free will and moral responsibility*. Oxford University Press.

4 For example, see: Anand, S., Fabienne P., & Amartya, S. (2004). *Public health, ethics, and equity*. Oxford University Press; Gostin, L. O., & Powers, M. (2006). What does social justice require for the public's health? Public health ethics and policy imperatives. *Health Affairs, 25*(4), 1053–1060; Persad, G., Wertheimer, A., & Emanuel, E. J. (2009). Principles for allocation of scarce medical interventions. *The Lancet, 373*(9661), 423–431; Wilkinson, D., Petrou, S., & Savulescu, J. (2018). Expensive care? Resource‐based thresholds for potentially inappropriate treatment in intensive care. *Monash Bioethics Review, 35*(1–4), 2–23; Harris, J. (1987). QALYfying the value of life. *Journal of Medical Ethics, 13*(3), 117–123.

5 See: Brown, R. C., & Savulescu, J. (2019). Responsibility in healthcare across time and agents. *Journal of Medical Ethics, 45*(10), 636–644; Davies, B., & Savulescu, J. (2019). Solidarity and responsibility in health care. *Public Health Ethics, 12*(2), 133–144; Pillutla, V., Maslen, H., & Savulescu, J. (2018). Rationing elective surgery for smokers and obese patients: Responsibility or prognosis? *BMC Medical Ethics, 19*(1), 28; Sharkey, K., & Gillam, L. (2010). Should patients with self‐inflicted illness receive lower priority in access to healthcare resources? Mapping out the debate. *Journal of Medical Ethics, 36*(11), 661–665; Savulescu, op. cit. note 2; Vallgårda, S., Nielsen, M. E. J., Hartlev, M., & Sandøe, P. (2015). Backward‐ and forward‐looking responsibility for obesity: Policies from WHO, the EU and England. *European Journal of Public Health, 25*(5), 845–848.

6 Although see: Ayres, P. J. (1996). Rationing health care: Views from general practice. *Social Science & Medicine, 42*(7), 1021–1025; Bringedal, B., & Feiring, E. (2011). On the relevance of personal responsibility in priority setting: A cross‐sectional survey among Norwegian medical doctors. *Journal of Medical Ethics, 37*, 357–361; Lauridsen, S. M., Norup, M., & Rossel, P. (2008). Bedside rationing by general practitioners: A postal survey in the Danish public healthcare system. *BMC Health Services Research, 8*(1),192; Perneger, T. V., Martin, D. P., & Bovier, P. A. (2002). Physicians' attitudes toward health care rationing. *Medical Decision Making, 22*(1), 65–70; Pinho, M., & Veiga, P. (2020). Attitudes of health professionals concerning bedside rationing criteria: A survey from Portugal. *Health Economics, Policy and Law, 15*(1), 113–127; Rosén, P., & Karlberg, I. (2002). Opinions of Swedish citizens, health‐care politicians, administrators and doctors on rationing and health‐care financing. *Health Expectations, 5*(2), 148–155; Ryynänen, O. P., Myllykangas, M., Kinnunen, J., & Takala, J. (1999). Attitudes to health care prioritization methods and criteria among nurses, doctors, politicians and the general public. *Social Science & Medicine, 49*(11), 1529–1539; Strech, D., Persad, G., Marckmann, G., & Danis, M. (2009). Are physicians willing to ration health care? Conflicting findings in a systematic review of survey research. *Health Policy, 90*(2–3), 113–124.

7 For example: Fischer & Ravizza, op. cit. note 3.

8 For example: Shoemaker, D. (2011). Attributability, answerability, and accountability: Toward a wider theory of moral responsibility. *Ethics, 121*(3), 602–632.

9 Vincent, N. A. (2011). A structured taxonomy of responsibility concepts. In N. A. Vincent, I. van de Poel, & J. van den Hoven (Eds.), *Moral responsibility: Beyond free will and determinism* (pp. 15–35). Springer, Springer Nature.

10 Earp, B. D., Demaree‐Cotton, J., Dunn, M., Dranseika, V., Everett, J. A., Feltz, A., Geller, G., Hannikainen, I. R., Jansen, L. A., Knobe, J., Kolak, J., Latham, S., Lerner, A., May, J., Mercurio, M., Mihailov, E., Rodríguez‐Arias, D., López, B. R., Savulescu, J., Sheehan, M., Strohminger, N., Sugarman, J., Tabb, K., & Tobia, K. (2020). Experimental philosophical bioethics. *AJOB Empirical Bioethics, 11*(1), 30–33; Nichols, S. (2006). Folk intuitions on free will. *Journal of Cognition and Culture, 6*(1–2), 57–86; Nahmias, E., Morris, S., Nadelhoffer, T., & Turner, J. (2005). Surveying freedom: Folk intuitions about free will and moral responsibility. *Philosophical Psychology, 18*(5), 561–584; Malle, B. F., & Knobe, J. (1997). The folk concept of intentionality. *Journal of Experimental Social Psychology, 33*(2), 101–121.

11 Foddy, B., & Savulescu, J. (2010). A liberal account of addiction. *Philosophy, Psychiatry, & Psychology: PPP, 17*(1), 1–22; Levy, N. (2006). Autonomy and addiction. *Canadian Journal of Philosophy, 36*(3), 427–447. https://doi.org/10.1353/cjp.2006.0018; Snoek, A. (2017). How to recover from a brain disease: Is addiction a disease, or is there a disease‐like stage in addiction? *Neuroethics, 10*(1), 185–194.

12 Broome, M. R., Bortolotti, L., & Mameli, M. (2010). Moral responsibility and mental illness: A case study. *Cambridge Quarterly of Healthcare Ethics, 19*, 179–187; King, M, & May, J. (2018). Moral responsibility and mental illness: A call for nuance. *Neuroethics, 11*(1), 11–22.

13 Bonnie, L. H., van den Akker, M., van Steenkiste, B., & Vos, R. (2010). Degree of solidarity with lifestyle and old age among citizens in the Netherlands: Cross‐sectional results from the longitudinal SMILE study. *Journal of Medical Ethics, 36*(12), 784–790.

14 Anderson, M., Richardson, J., McKie, J., Iezzi, A., & Khan, M. (2011). The relevance of personal characteristics in health care rationing: What the Australian public thinks and why. *American Journal of Economics and Sociology, 70*(1), 131–151.

15 Berk, M. L., Gaylin, D. S., & Schur, C. L. (2006). Exploring the public's views on the health care system: A national survey on the issues and options. *Health Affairs, 25*(6), w596–w606.

16 Miraldo, M., Galizzi, M., Merla, A., Levaqqi, R., Schulz, P., Auxilia, F., Castaldi, S., & Gellati, U. (2014). Should I pay for your risky behaviours? Evidence from London. *Preventive Medicine, 66*, 145–158.

17 Werntoft, E., & Edberg, A. K. (2009). Decision makers' experiences of prioritisation and views about how to finance healthcare costs. *Health Policy, 92*(2–3), 259–267.

18 Björk, J., Juth, N., & Lynøe, N. (2018). ‘Right to recommend, wrong to require’ – An empirical and philosophical study of the views among physicians and the general public on smoking cessation as a condition for surgery. *BMC Medical Ethics, 19*(1), 2.

19 Bringedal & Feiring, op. cit. note 6.

20 Pinho, M., & Borges A. P. (2019). The views of health care professionals and laypersons concerning the relevance of health‐related behaviors in prioritizing patients. *Health Education & Behavior, 46*(5),728–736.

21 Miraldo et al., op. cit. note 16.

22 Bringedal & Feiring, op. cit. note 6; Pinho & Borges, op. cit. note 20.

23 Ibid.

24 For example see Pillutla et al., op. cit. note 5.

25 Cost‐effectiveness of an intervention or treatment is determined by calculating how many quality‐adjusted life years (QALYs) it is expected to accrue for a given amount of spending. A QALY is a measure used to express the value of health states in order to compare the benefits of treatment. It is the arithmetic product of life expectancy and the quality of the remaining years. One QALY is equal to 1 year of life in perfect health, and QALYs are calculated by estimating the years of life remaining for a patient following a particular treatment or intervention and weighting each year with a quality‐of‐life score (on a 0–1 scale).

26 Harris, J. (2005). It's not NICE to discriminate. *Journal of Medical Ethics, 31*(7), 373–375.

27 Note that in the most up‐to‐date Norwegian legislation the severity criterion has been removed, though it was in place at the time of the surveys.

28 For example, see: The Health Systems and Policy Monitor. (2021, January 13). Health Systems in Transition (HiT) profile of Norway. https://www.hspm.org/countries/norway08012014/countrypage.aspx

29
https://assets.publishing.service.gov.uk/government/uploads/system/uploads/attachment_data/file/480482/NHS_Constitution_WEB.pdf

30 For a discussion see Pillutla et al., op. cit. note 5.

31 Royal College of Surgeons of England. (2016, April 22). *More than one in three areas of England restrict surgery for smokers and overweight patients* [Press release]. https://www.rcseng.ac.uk/news-and-events/media-centre/press-releases/more-than-one-in-three-areas-of-england-restrict-surgery-for-smokers-and-overweight-patients/

32 For example: Donnelly, L. (2018, March 18). Refusing surgery to obese people and smokers is ‘discriminatory and cruel’, surgeons say. *The Telegraph*. https://www.telegraph.co.uk/news/2018/03/02/refusing-surgery-obese-people-smokers-discriminatory-cruel-surgeons/; Rawlinson, K., & Johnston, C. (2016, September 3). Decision to deny surgery to obese patients is like ‘racial discrimination’. *The Guardian*. https://www.theguardian.com/society/2016/sep/03/hospitals-to-cut-costs-by-denying-surgery-to-smokers-and-the-obese

33 NHS Commissioning Board. (2013). *Commissioning policy: Ethical framework for priority setting and resource allocation*. https://www.england.nhs.uk/wp-content/uploads/2013/04/cp-01.pdf

34 Bringedal, B., Fretheim, A., Nilsen, S., & Rø, K. I. (2019). Do you recommend cancer screening to your patients? A cross‐sectional study of Norwegian doctors. *BMJ Open, 9*(8), e029739.

35 Bringedal & Feiring, op. cit. note 6; ibid.

36 Kahane, G., Everett, J. A., Earp, B. D., Caviola, L., Faber, N. S., Crockett, M. J., & Savulescu, J. (2018). Beyond sacrificial harm: A two‐dimensional model of utilitarian psychology. *Psychological Review, 125*(2), 131.

37 Pinho & Borges, op. cit. note 20.

38 Bringedal & Feiring, op. cit. note 6.

39 Pinho & Borges, op. cit. note 20.

40 Miraldo et al., op. cit. note 16.

41 Anderson et al., op. cit. note 14.

42 Brown & Savulescu, op. cit. note 5; Davies & Savulescu, op. cit. note 5; Pillutla et al., op. cit. note 5; Sharkey & Gillam, op. cit. note 5; Savulescu, op. cit. note 2; Vallgårda et al., op. cit. note 5.

43 See: Brown, R. C. (2013). Moral responsibility for (un) healthy behaviour. *Journal of Medical Ethics, 39*(11), 695–698.

44 See Brown, R. C., Maslen, H., & Savulescu, J. (2019). Against moral responsibilisation of health: Prudential responsibility and health promotion. *Public Health Ethics, 12*(2), 114–129.

45 Savulescu, op. cit. note 2.

46 Ibid.

47 For example, see: Véliz, C., Maslen, H., Essman, M., Taillie, L. S., & Savulescu, J. (2019). Sugar, taxes, & choice. *Hastings Center Report, 49*(6), 22–31.

48 It could also be that doctors thought that one is relatively unlikely to die from cutting one's wrists.

49 See: Brown et al., op. cit. note 44; Brown, R. C. H., Maslen, H., & Savulescu, J. (2019). Responsibility, prudence and health promotion. *Journal of Public Health, 41*(3), 561–565.

50 See: Davies & Savulescu, op. cit. note 5.

51 Bringedal & Feiring, op. cit. note 6; Bringedal et al., op. cit. note 34.

